# Controlling the corrosion and hydrogen gas liberation inside lead-acid battery via PANI/Cu-Pp/CNTs nanocomposite coating

**DOI:** 10.1038/s41598-021-88972-4

**Published:** 2021-05-04

**Authors:** M. A. Deyab, Q. Mohsen

**Affiliations:** 1 Egyptian Petroleum Research Institute (EPRI), PO Box 11727, Nasr City, Cairo, Egypt; 2 Department of Chemistry, College of Sciences, Taif University, Taif, Saudi Arabia

**Keywords:** Chemistry, Electrochemistry, Materials science

## Abstract

The liberation of hydrogen gas and corrosion of negative plate (Pb) inside lead-acid batteries are the most serious threats on the battery performance. The present study focuses on the development of a new nanocomposite coating that preserves the Pb plate properties in an acidic battery electrolyte. This composite composed of polyaniline conductive polymer, Cu-Porphyrin and carbon nanotubes (PANI/Cu-Pp/CNTs). The structure and morphology of PANI/Cu-Pp/CNTs composite are detected using transmission electron microscopy (TEM), scanning electron microscopy (SEM) and X-ray diffraction (XRD) analysis. Based on the H_2_ gas evolution measurements and Tafels curves, the coated Pb (PANI/Cu-Pp/CNTs) has a high resistance against the liberation of hydrogen gas and corrosion. Electrochemical impedance spectroscopy (EIS) results confirm the suppression of the H_2_ gas evolution by using coated Pb (PANI/Cu-Pp/CNTs). The coated Pb (PANI/Cu-Pp/CNTs) increases the cycle performance of lead-acid battery compared to the Pb electrode with no composite.

## Introduction

Indeed after 150 a long time since lead-acid battery (LAB) innovation, advancements are still being made to the lead battery performance and in spite of its inadequacies and the competition from more energy storage cells; the LAB battery still holds the lion's share of the total battery sales^1^.

In brief, in the LAB battery the PbO_2_ (positive plate) and Pb (negative plate) respond with the electrolyte (H_2_SO_4_) to form energy^2,3^. The main advantages of LAB battery are low cost, low internal impedance, and easily recycled^4^.

One of the most important difficulties facing the LAB battery industry is the liberation of bubbles of hydrogen gas and corrosion of negative plate (pb)^5–7^. This may cause a great low in battery performance and also explosion in the LAB battery room.

The utilize of added substances (additives) within the battery electrolyte is one of the approaches which offers an increase in battery performance without much modification of other components^8–11^. The major issue lies with choosing a reasonable added substance which is chemically, thermally and electrochemically steady in exceedingly corrosive environment.

To resolve these difficulties experimentally, many researchers tried to decrease the rate of the hydrogen gas (HER) and corrosion of negative plate (pb) by applying additives such as organic compounds, surfactants and ionic liquids^12–16^. These additives are utilized to increase performance of the LAB battery through working as cathodic-type inhibitors.

Previous research has shown that the use of conductive polymer coatings may be a good solution to overcome the failure in battery electrodes^17,18^. Unfortunately, the stability of conductive polymers under ambient conditions is a persistent problem. To maximize the efficiency of conductive polymer coatings, the different nano-particles with unique properties such as Cu-Porphyrins (Cu-Pp) and carbon nanotubes (CNTs) will be incorporated in the texture of PANI forming new nanocomposite coating.

The low cost, ease of synthesis, high environmental reliability, and high conductivity of PANI, Cu-Pp and CNTs make them promising materials for the formation of new composites^19,20^.

The composites containing nano-particles with conductive polymer PANI had been used for enhancing the battery performance in our previous studies^21,22^, but here we will develop for first-time novel composites containing three components (i.e. PANI, Cu-Pp and CNTs). Therefore, here we introduce a new strategy to protect a negative plate (pb) of LAB battery by developing a new nanocomposite coating PANI/Cu-Pp/CNTs that preserve the Pb plate properties in an acidic electrolyte.

## Materials and methods

### Materials

The negative plate of LAB battery was made from pure lead (Pb) 99.99%.

Cu-Porphyrin (5,10,15,20-Tetrakis-(4-aminophenyl)-porphyrin-Cu-(II)) was supplied from Service Chemical Inc.

PANI polymer was provided by SigmaeAldrich Co. CNTs were prepared in EPRI Lab. (number of layers ≈ 5–20; tube diameter ≈ 20–30 nm, tube length ≈ 1–10 mm). Battery electrolyte (5.0 M H_2_SO_4_ solution) was prepared from of AR grade 98% H_2_SO_4_ (Sigma–Aldrich).

Dimethylformamide (DMF) and N-methyl- pyrrolidone were purchased from Sigma–Aldrich.

### Preparation of PANI/Cu-Pp/CNTs and coated electrodes

Solution mixing is a simple and efficient approach used for the preparation of PANI/Cu-Pp/CNTs nanocomposite. The method includes the mechanical mixing followed by grinding of 1.0 g PANI, 0.2 g CNTs and 0.02 g Cu-Pp. The final powder was dispersed in 10.0 ml dimethylformamide (DMF) using ultrasonication for 60 min. The PANI/Cu-Pp/CNTs nanocomposite was obtained after the drying treatment to remove the solvent.

PANI/Cu-Pp/CNTs coatings were prepared by mixing PANI/Cu-Pp/CNTs nanocomposite powder and N-methyl- pyrrolidone (solvent) using ultrasonication tool at a frequency of 30 kHz for 60 min. The prepared coatings were applied on the surface of the negative plate of LAB battery by coating spray gun. The coated electrodes were dried at 343 K for 6.0 h. For comparison, the neat PANI and PANI/CNTs coatings were prepared by the same conditions.

### Methods

The experimental setup for the H_2_ gas evolution measurements was described in our earlier work^23,24^. For this purpose, the Pb electrodes (dimension = 1.5 cm × 0.5 cm × 0.04 cm) were placed in 5.0 M H_2_SO_4_ solution (100 ml). The period of immersion is 5 h. The rate of hydrogen evolution (HER) is calculated by dividing the volume of the hydrogen evolved (Δ*V*) to immersion time (*t*) and electrode surface area (*A*), as given in Eq. (1)^25^:

1 $$ {\text{HER }}\left( {{\text{ml\;min}}^{{ - {1}}} {\text{cm}}^{{ - {2}}} } \right) \, = \, \Delta{V}$$

Electrochemical tests (Tafel and EIS) were performed using glass cell (3-electrodes cell). The electrochemical responses were observed using Potentiostat instrument (model: Gill AC -947- ACM). In this system, the Pt and Hg/Hg_2_SO_4_ electrodes serve as counter and reference electrodes, respectively. The EIS experiment was performed in a frequency range 1.0 Hz–30 kHz at −1.1 V vs. Hg/Hg_2_SO_4_. The Tafel experiment was performed in a potential range (−250 mV) to (250 mV) versus OCP with short scan rate (1.0 mV s^−1^).

AC electrical conductivity of PANI, PANI/CNTs and PANI/Cu-Pp/CNTs were determined by impedance analyzer in frequency range 10 Hz–1000 kHz.

The cycle performance of LAB battery was inspected by using laboratory made cells (2.0 V/2.8 Ah). This cell contains one negative electrode and two positive electrodes. The separator is poly vinyl chloride. The electrolyte is 5.0 M H_2_SO_4_. The cycle performance tests were carried out using different negative electrodes i.e. bare Pb, coated Pb (neat PANI), coated Pb (PANI/CNTs) and coated Pb (PANI/Cu-Pp/CNTs). In all cases, the tests were stopped at 1.7 V (the cut-off discharging voltage) and measured at C/5 rate and at 298 K.

The structure and morphology of PANI/Cu-Pp/CNTs composite were detected using TEM, SEM (Jeol-Jem 1200EX II) and XRD (PANIalytical X'PERT PRO) analysis.

## Results and discussion

### Structure and morphology of PANI/Cu-Pp/CNTs

**Figure 1:**
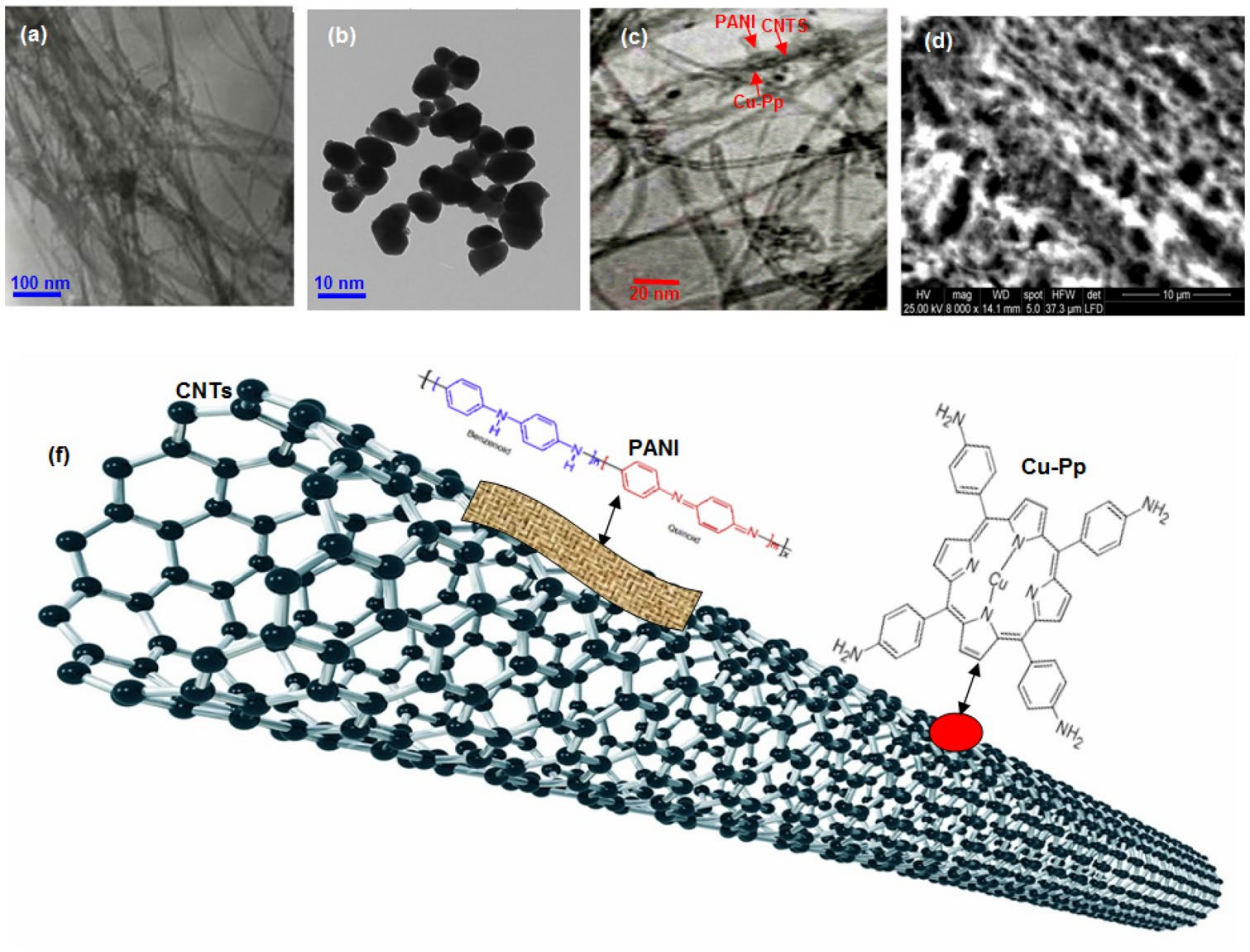
(**a**) TEM image of CNTs, (**b**) TEM image of Cu-Pp, (**c**) TEM image of PANI/Cu-Pp/CNTs composite, (**d**) SEM image of PANI/Cu-Pp/CNTs composite, and (**f**) The illustration of PANI/Cu-Pp/CNTs composite.

**Figure 2:**
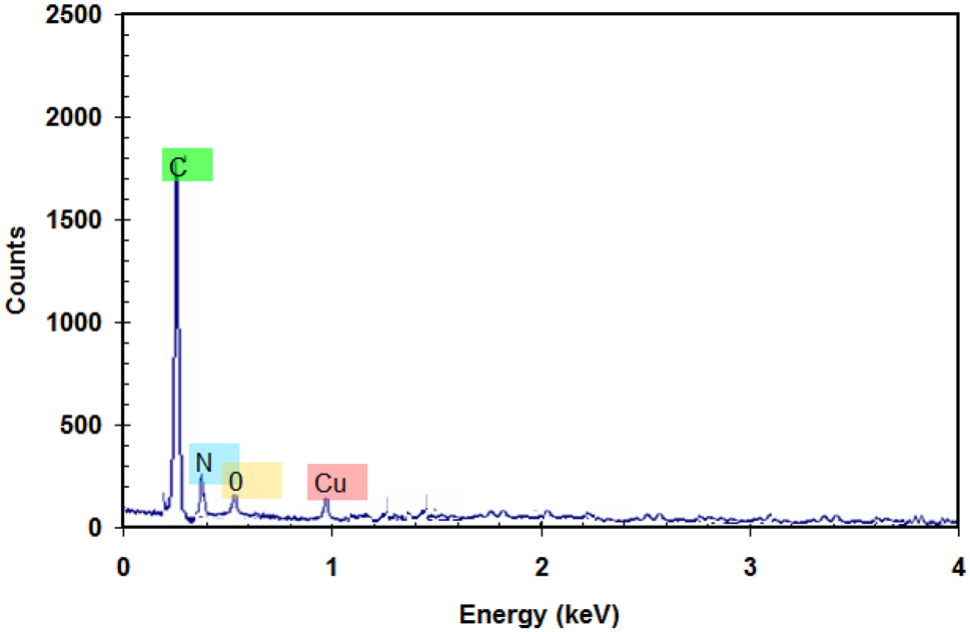
EDX analysis of PANI/Cu-Pp/CNTs composite.

TEM and SEM were conducted on the surface of the PANI/Cu-Pp/CNTs nanocomposite to detect the morphology of nanocomposite, as shown in Fig. 1. The relevant TEM image of CNTs can be seen in Fig. 1a, which illustrates that CNTs are made up of homogeneous tubes. The TEM image in Fig. 1b shows that the Cu-Pp particles have a nanoplate shape.The TEM images in Fig. 1c indicated that PANI/Cu-Pp/CNTs composite comprised both PANI layer on the surface of the CNTs wall and uniformly dispersed Cu-Pp particles on the CNTs. The homogeneous texture of PANI/Cu-Pp/CNTs was also confirmed by SEM image in the Fig. 1d. The illustration of this composite is shown in Fig. 1f. The presence of C, N, O, and Cu peaks in the EDS pattern (see Fig. 2) confirms the chemical composition of the PANI/Cu-Pp/CNTs composite.

**Figure 3:**
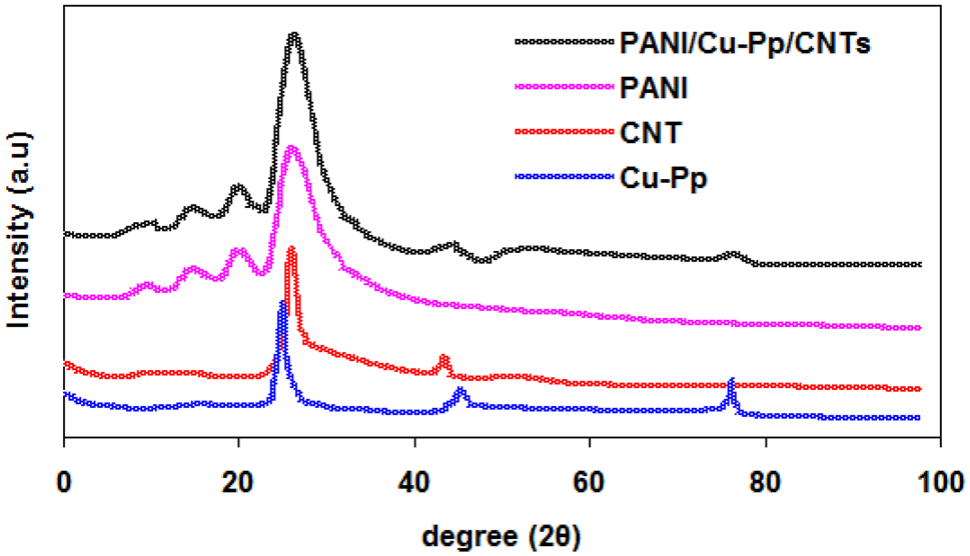
XRD patterns for neat PANI, CNTs, Cu-Pp and composite PANI/Cu-Pp/CNTs.

The XRD patterns for neat PANI, CNTs, Cu-Pp and composite PANI/Cu-Pp/CNTs are shown in Fig. 3. PANI has peaks at 2θ = 9.42$^\circ$, 14.70$^\circ$, 20.52$^\circ$, 25.52$^\circ$. CNTs has peaks at 2θ = 25.96$^\circ$, 42.5$^\circ$. Cu-Pp has peaks at 2θ = 24.83$^\circ$, 45.22$^\circ$, 76.20$^\circ$.

Characteristic peaks of PANI, CNTs and Cu-Pp can be seen in PANI/Cu-Pp/CNTs nanocomposite pattern. This confirms the formation of PANI/Cu-Pp/CNTs nanocomposite.

### Effects of PANI/Cu-Pp/CNTs on the hydrogen gas evolution

**Figure 4:**
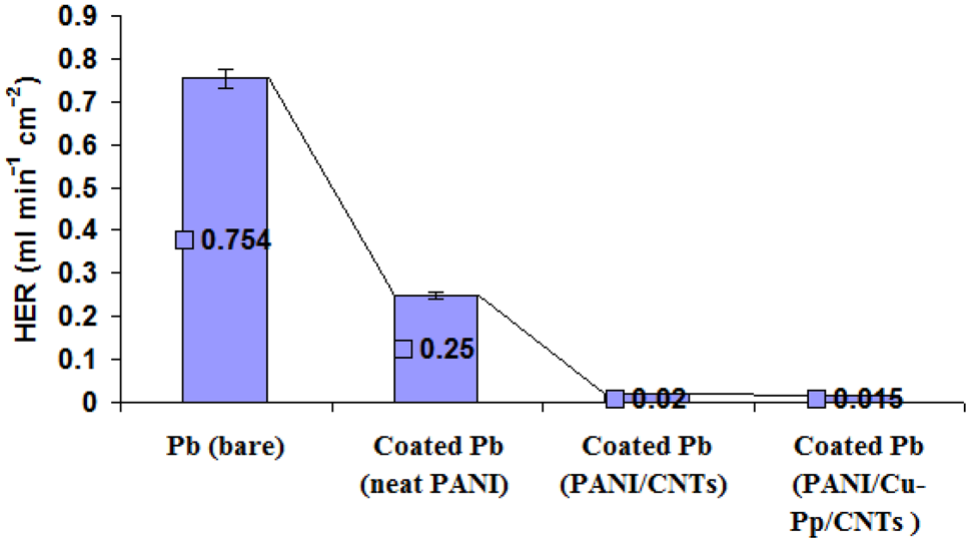
Hydrogen gas evolution (HER) for bare Pb, coated Pb (neat PANI), coated Pb (PANI/CNTs) and coated Pb (PANI/Cu-Pp/CNTs ) in 5.0 M H_2_SO_4_ at 298 K.

Figure 4 displays the hydrogen gas evolution results for bare Pb, coated Pb (neat PANI), coated Pb (PANI/CNTs) and coated Pb (PANI/Cu-Pp/CNTs ) in 5.0 M H_2_SO_4_ in order to determine the effects of new nanocomposites on the HER. In the case of bare Pb, HER was 0.754 ml min^−1^ cm^−2^. In comparison, coated Pb (neat PANI), coated Pb (PANI/CNTs) and coated Pb (PANI/Cu-Pp/CNTs ) revealed the HER around 0.25 ml min^−1^ cm^−2^, 0.02 ml min^−1^ cm^−2^ and 0.015 ml min^−1^ cm^−2^, respectively.

It is evident that new nanocomposites suppressed the hydrogen gas evolution reaction. Also, the considerable low HER was detected in coated Pb (PANI/Cu-Pp/CNTs).

**Figure 5:**
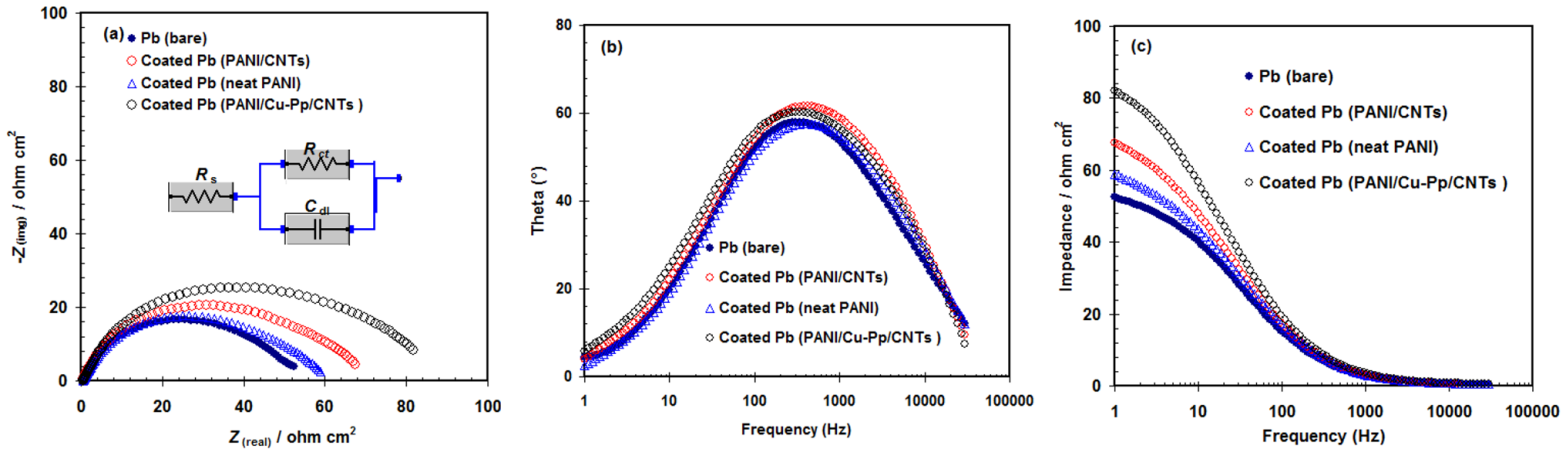
EIS plots (Nyquist (**a**), Bode (**b**) and Phase angle (**c**) plots) for bare Pb, coated Pb (neat PANI), coated Pb (PANI/CNTs) and coated Pb (PANI/Cu-Pp/CNTs ) in 5.0 M H_2_SO_4_ at − 1.1 V vs. Hg/Hg_2_SO_4_ and 298 K.

The HER for coated and uncoated Pb can be investigated via EIS experiments at − 1.1 V vs. Hg/Hg_2_SO_4_. Then, the EIS method can be used for extracting the impedance parameters for cathodic reaction (i.e. Hydrogen gas evolution reaction)^26^. Figure 5 shows the EIS plots (Nyquist (a), Bode (b) and Phase angle (c) plots) for bare Pb, coated Pb (neat PANI), coated Pb (PANI/CNTs) and coated Pb (PANI/Cu-Pp/CNTs ) in 5.0 M H_2_SO_4_ at − 1.1 V vs. Hg/Hg_2_SO_4_.

The Nyquist parts exhibit a similar trend (i.e. charge transfer trend) for all uncoated and coated electrodes^27^. The best suitable equivalent circuit (EC) for Nyquist parts was inserted in Fig. 5a. This EC contains *R*_ct_ (charge transfer resistance of HER on Pb), *C*_dl_ (electrical double layer capacitor) and *R*_s_ (electrolyte resistance)^28,29^. All these elements are presented in Table 1. Compared with the bare Pb, the coated electrodes with various coatings (neat PANI, PANI/CNTs and PANI/Cu-Pp/CNTs) showed higher *R*_ct_ and lower *C*_dl_ (see Table 1). The coated Pb (PANI/Cu-Pp/CNTs) had a relatively larger *R*_ct_ than that of the coated Pb (neat PANI) and the coated Pb (PANI/CNTs). Results confirmed that PANI/Cu-Pp/CNTs nanocomposite caused a decrease in the hydrogen gas evolution for Pb in 5.0 M H_2_SO_4_.

**Table 1:** Equivalent circuit elements of bare and coated Pb electrodes.

Electrode	*R*_s_ (Ω cm^2^)	*R*_ct_ (Ω cm^2^)	*C*_dl_ (F cm^−2^)
Bare Pb	0.72	45.77	3.52 × 10^–4^
coated Pb (neat PANI)	0.53	58.90	2.75 × 10^–4^
coated Pb (PANI/CNTs)	0.35	67.83	2.13 × 10^–4^
coated Pb (PANI/Cu-Pp/CNTs )	0.28	83.43	1.86 × 10^–4^

The decreased in the *R*_s_ values from 0.72 Ω cm^2^ to 0.28 Ω cm^2^ was observed for the coated Pb (PANI/Cu-Pp/CNTs). This could be assigned to the increase in the ions mobility through the composite layer^30^.

### Effects of PANI/Cu-Pp/CNTs on the corrosion rate

**Figure 6:**
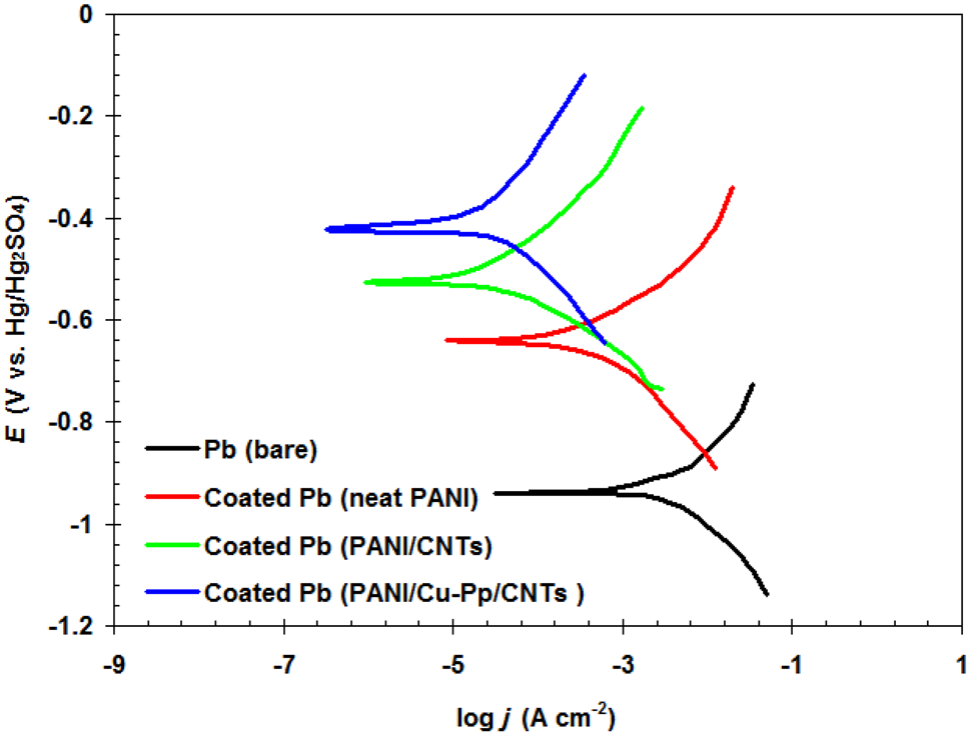
Tafel curves for bare Pb, coated Pb (neat PANI), coated Pb (PANI/CNTs) and coated Pb (PANI/Cu-Pp/CNTs ) in 5.0 M H_2_SO_4_ at 298 K.

Tafel experiments were used to examine the corrosion rate for various electrodes containing bare Pb, coated Pb (neat PANI), coated Pb (PANI/CNTs) and coated Pb (PANI/Cu-Pp/CNTs ) in 5.0 M H_2_SO_4_ (see Fig. 6). The Tafel elements such as corrosion potential (*E*_corr_) and corrosion current density (*j*_corr_) are presented in Table 2.

**Table 2:** The Tafel elements of bare and coated Pb electrodes.

Electrode	*j*_corr_ (mA cm^−2^)	*E*_corr_ (V vs. Hg/Hg2SO4)
Bare Pb	5.01	−0.942
coated Pb (neat PANI)	1.99	−0.640
coated Pb (PANI/CNTs)	0.14	−0.527
coated Pb (PANI/Cu-Pp/CNTs )	0.04	−0.421

Indeed, the new nanocomposites affect on the Tafel lines (anodic and cathodic reactions) for Pb in 5.0 M H_2_SO_4_. Compared with the bare Pb, the coated electrodes with various coatings (neat PANI, PANI/CNTs and PANI/Cu-Pp/CNTs) showed lower *j*_corr_ (see Table 2).

Results indicated that the *j*_corr_ decreased from 5.01 mA cm^−2^ to 0.04 mA cm^−2^ when the coated Pb (PANI/Cu-Pp/CNTs) was used. Furthermore, the *E*_corr_ moved to more positive direction for coated Pb electrodes. The change in *E*_corr_ values reflects a change in a corrosion system^31^. Results confirmed that PANI/Cu-Pp/CNTs nanocomposite caused a significant reduction in the corrosion rate for Pb in 5.0 M H_2_SO_4_. Furthermore, both the cathodic and anodic branches of the Tafel curves have shifted to lower current density values, suggesting that both hydrogen evolution and Pb dissolution reactions have been inhibited.

AC electrical conductivity experiments were used to prove that the low *j*_corr_ for coated Pb is not due to the poor conductance of nanocomposites.

In comparison, the AC conductivity of the PANI, PANI/CNTs and PANI/Cu-Pp/CNTs are 1.9 × 10^–8^ S cm^−1^, 3.5 × 10^–4^ S cm^−1^ and 9.2 × 10^–3^ S cm^−1^, respectively. In this case, CNTs may form bridge bonds with PANI, leading to the enhancement of the conductivity in the composite layers^32,33^.

In comparison, PANI/Cu-Pp/CNTs have the highest conductivity due to the fast electron delocalization along the PANI because of the combination of Zn-Pp and CNTs^34–37^.

### LAB battery performances

**Figure 7:**
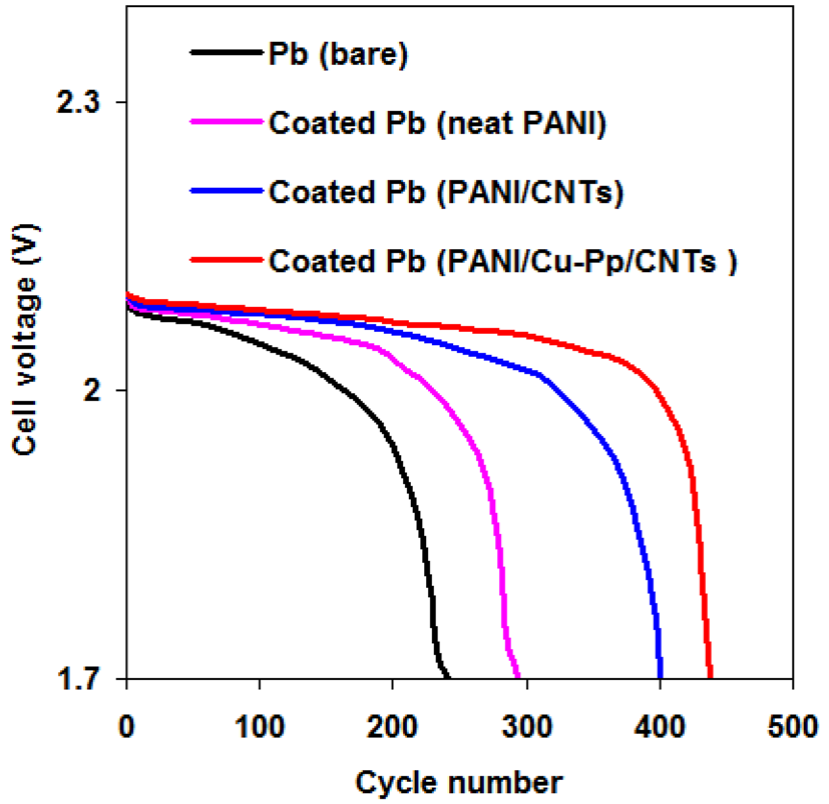
Dependence of cell voltage on cycle numbers using different negative electrodes.

Battery performances of LAB battery using different negative electrodes i.e. bare Pb, coated Pb (neat PANI), coated Pb (PANI/CNTs) and coated Pb (PANI/Cu-Pp/CNTs) were examined. The cycle performance of LAB battery was recorded in Fig. 7. The LAB battery showed an open circuit potential nearly 2.10 V. All electrodes showed the depleting in discharge voltage with an increase in cycle number. Here the discharge voltage at 1.7 V represents the battery shortage and the end of discharge.

In Fig. 7, the cycle numbers using various electrodes containing bare Pb, coated Pb (neat PANI), coated Pb (PANI/CNTs) and coated Pb (PANI/Cu-Pp/CNTs) are 234, 293, 400 and 438, respectively. Results displayed that coated Pb (PANI/Cu-Pp/CNTs) (negative electrode) gave the supreme performance comparing with bare Pb.

### Mechanism and explanation

When discharging a LAB battery, the following reactions at the negative electrode occurs: Pb + H_2_SO_4_ ↔ PbSO_4_ + 2H^+^  + 2e and 2H^+^  + 2e ↔ H_2_^38,39^. Hydrogen evolution and formation of PbSO_4_ on the surface of the negative electrode can induce the loss in the battery life^40,41^.

With regards to the above results, it is clear that the using of coated negative electrodes with PANI/Cu-Pp/CNTs composite can significantly decrease the HER and corrosion rates.

The most important mechanisms with respect to the role of PANI/Cu-Pp/CNTs composite can be based on the following aspects:

1. PANI polymer can form the physical barrier on the surface of Pb electrode. This barrier protects the surface of electrode from corrosive solution^42,43^.
2. Due to the conductivity property of the PANI polymer, the cathodic reaction (i.e. hydrogen evolution) that occurred on the surface of Pb electrode will be replaced with the PANI /electrolyte interface^44^. Therefore, we noted a significant reduction in the hydrogen evolution.
3. According to Ahmad and MacDiarmid^45^, the coating of PANI causes the moving of corrosion potential to the passive area, leading to protection of Pb electrode. Additionally, this moving in *E*_corr_ gives a significant physical property against corrosion products on the Pb electrode^46^.
4. By introducing the CNTs and Cu-Pp nanoparticles in the PANI polymer matrix, that formed composite (PANI/Cu-Pp/CNTs) becomes more effective in the suppression of both hydrogen gas evolution and corrosion of Pb electrode than PANI alone^22,34^.
5. The presence of CNTs and Cu-Pp nano-particles shrink the electrolyte pathway of PANI and hence reducing the risks of corrosive solution. Moreover, both nanoparticles improve the mechanical and conductivity properties of PANI^47–50^.
6. The use of PANI/Cu-Pp/CNTs composite was effective for reducing the formation of PbSO_4_ on the surface of the battery negative electrode during the cycling process. This led to the supreme performance of the LAB battery.
7. The high conductivity of CNTs and Cu-Pp nanoparticles enhanced the cycling performance of LAB battery^51–53^.

## Conclusions

In the research, we have offered the promising composite (PANI/Cu-Pp/CNTs) coating to protect the negative plate (Pb) of LAB battery. PANI/Cu-Pp/CNTs nanocomposite was compared with neat PANI, PANI/CNTs coatings to determine the performance of new nanocomposite. In comparison, coated Pb (neat PANI), coated Pb (PANI/CNTs) and coated Pb (PANI/Cu-Pp/CNTs) revealed the HER around 0.25 ml min^−1^ cm^−2^, 0.02 ml min^−1^ cm^−2^ and 0.015 ml min^−1^ cm^−2^, respectively. Compared with the bare Pb, the coated Pb electrodes showed higher *R*_ct_ and lower *C*_dl_ and *I*_corr_. The presence of CNTs and Cu-Pp nano-particles improve the mechanical and conductivity properties of PANI. The coated Pb (PANI/Cu-Pp/CNTs) presented a better cyclic performance compared with bare Pb electrode. This means that the use of composite (PANI/Cu-Pp/CNTs) is effective coating to enhance the performance of LAB battery. This outcome opens up magnificent opportunities for nanocomposite research that is applied to lead-acid batteries.
